# Dr. Syer, on Delivering the Placenta

**Published:** 1800-07

**Authors:** J. Syer

**Affiliations:** Melton, Suffolk


### To the Editors of the Medical and Phyfical Journal.

GENTLEMEN,

THE pleafure and information derived by the Faculty from your very interefting periodical publication, merits their acknowledgement; and as opinions will fometimes very manifeftly differ with refpect to the treatment of particular cafes, and occafionally with propriety, yet, although candour impofes your filence, and in fome fort prevents your animadverfions, a difcriminating public will approve or cenfure fuch as militate againft the foundeft practice, and beft authorities: Through the fame medium, I beg leave to offer an opinion refpecting the manual or forcible delivery of the placenta, fo ftrongly recommended and enforced by feveral profeffional gentlemen in two or three of your laft Medical Journals.

Having practifed midwifery for full twenty-five years upon a very extenfive fcale, and, I flatter myfelf, with as much fuccefs as moft men, during which period a variety of cafes have prefented themfelves, wherein fuch a practice *might* have been thought expedient; but, from long experience, I am taught to believe firmly, that in nineteen cafes out of twenty, more danger and mifchief would be induced than obviated by fuch attempts, rather than by fubmitting the bufinefs to Nature.

The following cafe, at an early period of my practice, awakened my apprehenfion of the danger of the manual extraction, and influenced my conduct ever after; except in the moft urgent cafe, as it *then* appeared. — I was called to a gentlewoman in labour of her third child, who had for three or four years been fubject to fainting fits, (not of the epileptic or convulfive kind) and had had them occafionally during her former labours, without any farther ill confequences than retarding their progrefs in fome degree, having always had long, flow, and very painful labours, though perfectly natural before. A prefentation of a portion of the funis in this cafe had taken place immediately upon the rupture of the membranes, before I reached my patient, at fome miles diftance from me, who had a midwife with her. Finding a good pulfation, I was induced to return the funis, which I effected, per digitale, (abfente dolore) and a fingle blade of the forceps, with which, in almoft every cafe of fuch prefentation, I have fucceeded, with perfect fafety to the mother and child: the labour advanced fafely, though flowly afterwards, and fhe was in eight or ten hours delivered of a fine living child; immediately almoft after which, a confiderable hæmorrhage enfued, but ceafed foon upon the application of cold vinegar and water and brandy, with an opiate, together with her fyncope, which had occurred twice before during her labour. Being extremely anxious for the fafety and prefervation of my patient, under the above preffing circumftances, upon her recruiting, an hour after finding no advance, by gentle efforts at the funis during little paroxyfms of pain, I was induced (as I then thought juftifiably), as well as by the intreaty of my patient and by-ftanders, to extract the placenta, (manus introductione) which in the moft cautious and gentle manner I feparated through the hour-glafs contraction of the uterus, as is ufual, with little or no effufion; but a few minutes after, a fudden, though apparently not profufe, hæmorrhage took place, which produced the ufual fyncope, from which fhe never recovered.

The unpleafant fuggeftion upon this occafion, that probably my patient might have furvived, if the bufinefs had been left to Nature, impreffed and reproached me for officious zeal.

The cafe quoted by Mr. Wagftaff, (NO. XV.) although it terminated fafely, yet, from the circumftances therein mentioned, it appeared officious, and would obvioufly have proved equally fo, if left to Nature, a few hours afterwards.

Mr. Davies's cafe (NO. XI.) clearly proves the non-neceffity likewife of manual or *forcible* delivery; for, if it is ever expedient, which *may* be the cafe, the moft cautious and gradual feparation ought to take place, not only to obviate hæmorrhage and inflammation, but the truly formidable mifchief of inverfio vel prolapfus uteri, which proves too frequently the confequence of the rough and rude hand of hafte and ignorance, more efpecially of half-informed women midwives, an occurrence that I have feveral times met with, to the great forrow and difcomfort of many a valuable woman ever after. How guarded, therefore, ought gentlemen to be, in recommending and fanctioning a practice pregnant with fo much mifchief.

Mr. Peck's advice (in NO. XIII.) feems ftill more exceptionable than the former, in recommending the practice of forcible extraction invariably, if a fpontaneous delivery does not fucceed in ten or fifteen minutes.

The fuccefsful termination of Dr. Scott's cafe, (NO. XV.) by no means juftified the hazardous attempts, in the extremely reduced ftate of his patient; more time given for her to recruit, an opiate, and frigid applications, would in a few hours, moft probably, have induced a fpontaneous feparation, as I have repeatedly experienced.

I was much pleafed to fee in your laft Number of the MEDICAL JOURNAL, fome very judicious and pertinent remarks upon upon this subject by Dr. Squire, a respectable practitioner and teacher of Midwifry, wherein he ably combated the arguments advanced by the foregoing advocates for the practice, and instanced a variety of cogent reasons, as well as the highest authorities, against it; at the same time I was about transmitting these remarks, annexing many of the truly respectable authorities he has quoted against the practice, which now it is needless to recapitulate, therefore I shall only beg leave to corroborate what he has advanced on the subject, as from considerable practice and observation I am perfectly convinced of the propriety and rectitude of his advice, and shall be happy to see his further future observations on the management of the placenta, &c. which he announces is his intention of giving to the public; and am,

GENTLEMEN,

With much respect,

Your obedient humble servant,

J. SYER.

*Melton, Suffolk,*
*May* 29, 1800.

